# Diagnostic Performance of Self‐Collected Respiratory Swabs for SARS‐CoV‐2 and Influenza Virus in Community‐Dwelling Older Adults

**DOI:** 10.1111/irv.70241

**Published:** 2026-02-26

**Authors:** Xinyi Li, Samuel M. S. Cheng, Yuyun Chen, Faith Ho, Gigi Liu, Alan Au, Natalie Yu, Dennis K. M. Ip, Malik Peiris, Benjamin J. Cowling, Nancy H. L. Leung

**Affiliations:** ^1^ WHO Collaborating Centre for Infectious Disease Epidemiology and Control, School of Public Health, Li Ka Shing Faculty of Medicine The University of Hong Kong Pokfulam Hong Kong Special Administrative Region China; ^2^ Centre for Immunology & Infection Hong Kong Science and Technology Park New Territories Hong Kong Special Administrative Region China; ^3^ Laboratory of Data Discovery for Health Limited Hong Kong Science and Technology Park New Territories Hong Kong Special Administrative Region China

**Keywords:** COVID‐19, diagnostic performance, influenza, sampling method, self‐swab

## Abstract

**Background:**

Reassurance over the quality of self‐collected swabs could provide additional evidence to support the self‐collection of respiratory specimens as a valid and efficient method in large‐scale cohort studies.

**Methods:**

This study assessed the diagnostic performance of self‐collected pooled nasal and throat swabs compared to staff‐collected swabs for detecting SARS‐CoV‐2 and influenza virus by RT‐PCR in a cohort of older adults (aged 69–87 years) from March to October 2024.

**Results:**

Self‐collected swabs demonstrated high sensitivity (95.2% for SARS‐CoV‐2, 100% for influenza A) and specificity (99.2% and 100%, respectively) against staff‐collected swabs. Cycle threshold values showed no statistically significant differences and strong correlations between the two methods.

**Conclusions:**

These findings suggest self‐collected swabs are a reliable alternative for community‐based respiratory viruses surveillance in older adults, supporting their use in large‐scale studies.

## 1 Background

Self‐collection of swabs has the potential to simplify community‐based respiratory virus research [1]. Home use of rapid antigen tests (RATs) was common for COVID‐19 but has been less common for influenza to date. Reassurance over the quality of self‐collected swabs could provide additional evidence to support the self‐collection of respiratory specimens as a valid and efficient method in large‐scale cohort studies.

The accuracy of self‐collected swabs for COVID‐19 has been validated across different populations, including healthcare workers (HCWs) [2], symptomatic patients [3], and adolescents [4]. For SARS‐CoV‐2 diagnosis, a systematic review identified three studies on pooled nasal and throat swabs [5], including only one study comparing self‐collected pooled nasal and throat swabs with HCW‐collected nasopharyngeal swabs [6]. For influenza, a meta‐analysis reported nine studies comparing self‐ and HCW‐collected swabs [7], with only one study specifically focusing on older adults seeking outpatient care [8]. Here, in an open‐label uncontrolled trial of influenza vaccination in community‐dwelling older adults, we compared the diagnostic performance of self‐collected pooled nasal and throat swabs against those collected by trained research staff for both SARS‐CoV‐2 and influenza virus.

## 2 Methods

### 2.1 Study Participants

In 2017/2018, we enrolled and randomized 1861 older adults aged 65–82 in a randomized trial of enhanced influenza vaccines [9]. All individuals still enrolled at the end of this randomized trial in September 2021 (i.e., at 69–87 years of age) were approached to enroll in a new prospective uncontrolled trial of annual influenza vaccination with Flublok (Sanofi Pasteur) for a further 4 years. Individuals were excluded if they showed signs of dementia or significant cognitive impairment and thus were not capable of providing informed consent, if they reported contraindications to inactivated influenza vaccine (e.g., severe allergies), or if they reported contraindications to intramuscular injection (e.g., bleeding disorders).

We collected baseline information including age, sex, marriage status, and education level. We initiated monitoring of acute respiratory illnesses (ARIs) by active surveillance on January 16, 2022. Home visits were arranged for participants if they reported (1) two or more ARI symptoms with onset within the prior 7 days and symptoms still present on the day of home visit or (2) a positive COVID‐19 test within the prior 7 days. Between March and October 2024, we arranged home visits to collect pooled nasal and throat swabs by trained research staff and separately by participants themselves. These paired samples were the focus of the present analyses. We also only included paired samples from ARI episodes with at least two reported ARI symptoms in our analyses.

During home visits, study staff introduced the standard procedure for the pooled nasal and throat swabs collection and collected samples. Participants confirmed they understood the procedure and collected another set of pooled nasal and throat swabs without any physical assistance provided by study staff. A nasal swab collection involved three to five rotations of the swab in each naris. A throat swab was collected by swabbing the tonsillar areas and the posterior nasopharynx without touching other areas of the oral cavity. Both nasal swab and throat swab were then snapped off into the same tube containing viral transport medium (5% bovine serum albumin in Earle's balanced salt solution with antibiotic). Samples were then immediately stored in a cool bag with at least two ice packs to maintain 2°C–8°C and transported to our laboratory within 24 h. Our study protocol was reviewed and approved by the Institutional Review Board of the University of Hong Kong. All study participants signed written informed consent.

### 2.2 Laboratory Methods

Pooled swab samples were tested by RT‐PCR for virologic confirmation of infection for SARS‐CoV‐2 and influenza virus, with human ribonuclease P (RNase P) as an internal control to confirm sample quality. For SARS‐CoV‐2, samples were first screened for the nucleocapsid (N) gene, and in positive samples, followed by testing for the ORF1b‐nsp14 segment to confirm infection. For influenza virus, samples were tested for the matrix (M) gene of influenza A/B virus to confirm infection, followed by H1/H3 subtyping of influenza A virus or genotyping of influenza B virus lineages. An RT‐PCR test with a Ct value of ≤ 40 was determined positive.

### 2.3 Statistical Analysis

Sensitivity and specificity were calculated using staff‐collected swabs as reference, with binomial 95% confidence intervals. The Pearson correlation coefficient was calculated to assess the strength of association between Ct values of self‐collected swabs and staff‐collected swabs. Ct values were compared between self‐collected swabs and staff‐collected swabs across all days since symptoms onset and separately in each individual day since symptoms onset for RNase P in all paired samples and viral target genes in paired samples with at least one positive result using the Kruskal–Wallis rank sum test. Data analyses were conducted using R version 4.4.1 (R Foundation for Statistical Computing, Vienna, Austria). Two‐tailed *p* values < 0.05 were considered statistically significant.

## 3 Results

We included in our analysis a total of 202 pairs of self‐collected and staff‐collected samples from ARI episodes of at least two symptoms collected from 174 participants from March to October 2024 (Figure S1), after excluding three paired samples from collection initiated by a positive COVID‐19 test only (one asymptomatic episode and two episodes with only one symptom). These 174 participants had a median baseline age of 74 years (interquartile range, IQR: 71–77) (Table S1). Of these, 22 participants had more than one ARI episode, including 21 participants who had no more than one infection with SARS‐CoV‐2 or influenza confirmed by at least one sample, and one infected with SARS‐CoV‐2 followed by influenza confirmed by paired samples. Eighty‐four participants (34 males and 50 females) with a median age of 74 years (IQR 72–78) tested positive for SARS‐CoV‐2 in at least one sample; similarly, 22 participants (12 males, 54.6%) with a median age of 74 years (IQR 71.25–76) tested positive for influenza A virus. For influenza A virus, 17 and 3 tested positive in at least one sample for H1 and H3, respectively, with no H1 and H3 co‐infection detected in the self‐swab nor staff‐swab. No influenza B virus infection was detected. The median days from symptom onset to sample collection was 4 days (IQR: 3–5) for SARS‐CoV‐2 and 3 days (IQR: 2.25–4) for influenza A virus.

**FIGURE 1:**
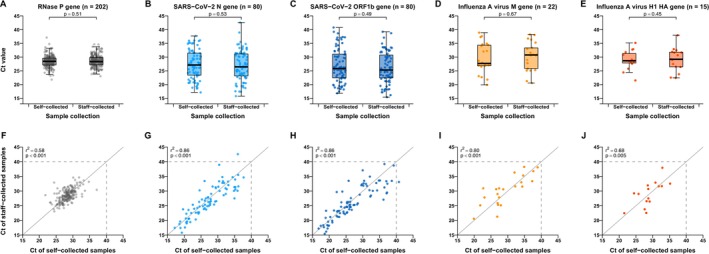
Comparison of Ct values in paired self‐collected and staff‐collected pooled nasal and throat swabs from older adults with acute respiratory illness associated with SARS‐CoV‐2 or influenza A virus infection. Sample pairs with detectable viral load in both self‐ and staff‐collected samples were shown. Boxplots of Ct values for self‐ and staff‐collected swabs (A–E) and linear correlation between self‐collected and staff‐collected swabs (F–J) were displayed by RNase P gene (internal control) (A, F), SARS‐CoV‐2 N gene (B, G), SARS‐CoV‐2 ORF1b gene (C, H), influenza A virus M gene (D, I), and influenza A virus H1 HA gene (E, J). For panels (A)–(E), Ct values between self‐ and staff‐collected swabs were tested by the Kruskal–Wallis rank sum test. For panels (F)–(J), linear correlation was shown by the Pearson correlation coefficient with the significance test, and dots below and to the left of the Ct ≤ 40 threshold (horizontal and vertical dashed lines) were determined to be positive. The diagonal reference dashed lines represent equality of Ct values from self‐collected and staff‐collected swabs.

All paired self‐collected and staff‐collected pooled nasal and throat swabs were valid collections as confirmed by the RNase P gene. The sensitivity of self‐collected swabs was 95.2% (95% confidence interval, CI: 88.1%–98.7%) and specificity was 99.2% (95% CI: 95.4%–100%) for SARS‐CoV‐2 and 100% (95% CI: 84.6%–100%) sensitive and 100% (95% CI: 98.0%–100%) specific for influenza A virus (Table S2). Ct values of self‐swabs and staff‐swabs were significantly highly correlated for each target gene for SARS‐CoV‐2 (0.86 for both N gene and ORF1b gene) and influenza A virus (0.80 for M gene and 0.68 for H1 HA gene) (Figure 1G–J). Ct values for self‐collected swabs and staff‐collected swabs did not show significant differences for any of the five genes (Figure 1A–E) or by collection day after symptoms onset (Figure S2).

## 4 Discussion

Our study found that self‐collection of pooled nasal and throat swabs is a valid and efficient approach for laboratory detection of symptomatic SARS‐CoV‐2 and influenza infections among older adults in the community setting in Hong Kong. Sample quality of self‐collected swabs was comparable to staff‐collected swabs (Figure 1F). We observed very high sensitivity and specificity of self‐collected swabs compared to staff‐collected swabs for both SARS‐CoV‐2 and influenza virus. Ct values of self‐swabs and staff‐swabs were highly correlated for each target gene for SARS‐CoV‐2 and influenza.

Consistent with previous studies reporting that almost all self‐collected swabs demonstrated adequate sampling quality [4, 8, 10, 11, 12], our study observed adequacy in all samples as evidenced by RNase P gene detection. A meta‐analysis reported comparable diagnostic performance for SARS‐CoV‐2 from pooled nasal and throat swabs (with nasopharyngeal swabs as reference) between studies using self‐collected samples (sensitivity 97%, specificity 100%) and those using HCW‐collected samples (sensitivity 97%, specificity 98%) [5]. For influenza virus, a study in Thailand among older adults seeking outpatient care for ARI reported 88% sensitivity and 100% specificity when comparing self‐collected versus staff‐collected nasal swabs [8]. The high diagnostic performance reported in our study could be attributed in part to the use of pooled swab instead of a single swab, demonstration of the swab collection procedure when staff‐collection was performed first, and participants' prior experience with self‐administered RAT during the COVID‐19 pandemic. Our order of collection was not pre‐specified, with staff‐collection performed first than self‐collection in 182 (90%) pairs. A study showed high concordance in RT‐PCR Ct values in sequentially collected mid‐turbinate nasal swabs for SARS‐CoV‐2 [13]. Future studies could randomize the collection order [14].

Our study supports self‐collection of pooled nasal and throat swabs as a valid alternative to staff‐collection for monitoring SARS‐CoV‐2 and influenza virus in large‐scale community‐based studies among older adults. Self‐collection of respiratory swabs has the potential to reduce the delay of sample collection by enabling participants to collect samples immediately upon recognizing symptoms [15]. However, self‐collection may encounter logistical challenges and might need to address biohazard issues associated with mailing potentially infectious samples.

## Author Contributions

**Xinyi Li:** data curation, formal analysis, visualization, writing – original draft, writing – review and editing. **Samuel M. S. Cheng:** investigation, writing – review and editing. **Yuyun Chen:** project administration, investigation. **Faith Ho:** data curation. **Gigi Liu:** investigation. **Alan Au:** investigation. **Natalie Yu:** investigation. **Dennis K. M. Ip:** writing – review and editing. **Malik Peiris:** supervision, writing – review and editing. **Benjamin J. Cowling:** supervision, writing – review and editing, funding acquisition. **Nancy H. L. Leung:** conceptualization, funding acquisition, methodology, supervision, writing – original draft, writing – review and editing. All authors reviewed and approved the final manuscript.

## Funding

The work described in this paper was supported by the Health and Medical Research Fund of the Hong Kong SAR Government (grant no. COVID1903001) and a grant from the Research Grants Council of the Hong Kong Special Administrative Region, China (project no. T11‐705/21‐N).

## Conflicts of Interest

B.J.C. has consulted for AstraZeneca, Fosun Pharma, GlaxoSmithKline, Haleon, Moderna, Novavax, Pfizer, Roche, and Sanofi Pasteur. All other authors declare no conflicts of interest.

## Supporting information


**Appendix S1:** Supporting information.

## Data Availability

The data that support the findings of this study are available from the corresponding author upon reasonable request.
