# Supplementary material for: Diagnostic Performance of Self‐Collected Respiratory Swabs for SARS‐CoV‐2 and Influenza Virus in Community‐Dwelling Older Adults
Source: Influenza Other Respir Viruses. 2026 Feb 26;20(3):e70241. doi: 10.1111/irv.70241 (PMC12946452; doi:10.1111/irv.70241)
Supplement: Supplementary file 1 — Appendix S1: Supporting information. [file IRV-20-e70241-s001.docx]

**Appendix Table 1. Baseline characteristics of 174 participants included in the final analysis.** For influenza A virus, 17 and 3 tested positive in at least one sample for H1 and H3, respectively, with no H1 and H3 co-infection. No influenza B virus infection was detected.

|  | **All participants** |  | **SARS-CoV-2** | | |  | **Influenza A virus** | | |
| --- | --- | --- | --- | --- | --- | --- | --- | --- | --- |
|  |  |  | **Positive*** | **Negative** | **P-value** |  | **Positive*** | **Negative** | **P-value** |
|  | **(n=174)** |  | **(n=84)** | **(n=90)** |  |  | **(n=22)** | **(n=152)** |  |
| **Age, median (IQR)** | 74 (71-77) |  | 74 (72-78) | 73 (71-76) | 0.10^a^ |  | 74 (71.25-76) | 74 (71-77) | 0.75^a^ |
| **Sex, No. (%)** |  |  |  |  | 0.16^b^ |  |  |  | 0.56^b^ |
| Male | 81 (46.6) |  | 34 (40.5) | 47 (52.2) |  |  | 12 (54.6) | 69 (45.4) |  |
| Female | 93 (53.4) |  | 50 (59.5) | 43 (47.8) |  |  | 10 (45.5) | 83 (54.6) |  |
| **Marriage status, No. (%)** |  |  |  |  | 0.81^b^ |  |  |  | 0.17^c^ |
| Married | 135 (77.6) |  | 64 (76.2) | 71 (78.9) |  |  | 20 (91.9) | 115 (75.7) |  |
| Separated/Widowed/Never married | 39 (22.4) |  | 20 (23.8) | 19 (21.1) |  |  | 2 (9.1) | 37 (24.3) |  |
| **Education level, No. (%)** |  |  |  |  | 0.72^c^ |  |  |  | 0.36^c^ |
| No schooling | 12 (6.9) |  | 7 (8.3) | 5 (5.6) |  |  | 3 (13.6) | 9 (5.9) |  |
| Primary school | 46 (26.4) |  | 22 (26.2) | 24 (26.7) |  |  | 6 (27.3) | 40 (26.3) |  |
| Secondary school or above | 115 (66.1) |  | 54 (64.3) | 61 (67.8) |  |  | 13 (59.1) | 102 (67.1) |  |
| Unknown or refused | 1 (0.6) |  | 1 (1.2) | 0 (0) |  |  | 0 (0) | 1 (0.7) |  |
| **Days since symptom onset,  median (IQR)** | 4 (3-5)^†^ |  | 4 (3-5) | 4 (3-5) | 0.53^a^ |  | 3 (2.25-4) | 4 (3-5) | 0.06^a^ |

* At least one of the staff-collected or self-collected swabs was positive.

† Days since symptoms onset calculated from 202 episodes reported by 174 participants.

a. P-value calculated by Kruskal-Wallis rank sum test

b. P-value calculated by Chi-squared test

c. P-value calculated by Fisher's exact test

**Appendix Table 2. Comparison between self- and staff-collected swabs for SARS-CoV-2 and influenza A virus detection by RT-PCR.** RT-PCR: real-time polymerase chain reaction

|  |  | **Staff-collected samples** | | | | | | |
| --- | --- | --- | --- | --- | --- | --- | --- | --- |
|  |  | **SARS-CoV-2** | | |  | **Influenza A virus** | | |
|  |  | **Positive** | **Negative** | **Total** |  | **Positive** | **Negative** | **Total** |
| **Self-collected samples** | **Positive** | 79 | 1 | 80 |  | 22 | 0 | 22 |
|  | **Negative** | 4 | 118 | 122 |  | 0 | 180 | 180 |
|  | **Total** | 83 | 119 | 202 |  | 22 | 180 | 202 |

**Appendix Figure 1. Timeline of SARS-CoV-2 and influenza A virus detections among older adults in the study, compared with population surveillance data in Hong Kong.** Panel A indicates the timeline of all paired sample collection (n=202), including negative detections for both SARS-CoV-2 and influenza A virus confirmed by both self- and staff-collected samples (n=96), at least one sample positive for SARS-CoV-2 (n=84), influenza A H1N1 (n=17), influenza A H3N2 (n=3) and unsubtyped influenza A virus (n=2), respectively. No influenza B virus infections were detected. Panel B shows the proportion of SARS-CoV-2 and influenza virus detections among all samples tested over time in population laboratory surveillance by Centre for Health Protection in Hong Kong.

**Appendix Figure 2. Differences in Ct values between self-collected and staff-collected swab by each day since symptoms onset for (A) human RNase P gene, (B) SARS-CoV-2 nucleocapsid or (C) ORF1b gene, (D) influenza A virus M gene and (E) influenza A(H1N1) virus hemagglutinin gene.** Positive value indicates Ct value for self-collected swab was higher than that of staff-collected swab. No statistically significant difference was detected by Kruskal-Wallis rank sum tests in Ct values for self-collected versus staff-collected swabs for each day since symptoms onset for any of the five genes.
